# Prognosis of Early-Onset *vs.* Late-Onset Mild Cognitive Impairment: Comparison of Conversion Rates and Its Predictors

**DOI:** 10.3390/geriatrics1020011

**Published:** 2016-04-25

**Authors:** Miguel Tábuas-Pereira, Inês Baldeiras, Diana Duro, Beatriz Santiago, Maria Helena Ribeiro, Maria João Leitão, Catarina Oliveira, Isabel Santana

**Affiliations:** 1 Neurology department, Centro Hospitalar e Universitário de Coimbra, Coimbra 3000-075, Portugal; hbmcsantiago@hotmail.com (B.S.); catarina.n.oliveira@gmail.com (C.O.); isabeljsantana@gmail.com (I.S.); 2 Neurochemistry laboratory, Neurology department, Centro Hospitalar e Universitário de Coimbra, Coimbra 3000-075, Portugal; ines.baldeiras@sapo.pt (I.B.); mhgarrucho@gmail.com (M.H.R.); jajao86@gmail.com (M.J.L.); 3 Center for Neuroscience and Cell Biology, Coimbra, Coimbra 3000-075, Portugal; 4 Neuropsychology unit, Neurology department, Centro Hospitalar e Universitário de Coimbra, Coimbra 3000-075, Portugal; diana.duro@gmail.com; 5 Faculty of Medicine, University of Coimbra, Coimbra 3000-548, Portugal

**Keywords:** mild cognitive impairment, Alzheimer, dementia, conversion, early-onset, late-onset, biomarkers, MCI

## Abstract

Background: Despite having the same histopathological characteristics, early-onset and late-onset Alzheimer’s disease (AD) patients show some distinct clinical and neuropsychological profiles. Early Onset Mild Cognitive Impairment (EOMCI) is a less characterized group. The aim of this study is to characterize MCI probably due to AD in terms of the clinical, genetic, Cerebrospinal fluid (CSF) biomarkers profile and conversion rate of EOMCI, compared to the late-onset form (LOMCI). Methods: 159 MCI patients were divided in two groups: 52 EOMCI (onset < 65 years) and 107 LOMCI (onset ≥ 65 years). We investigated differences in neuropsychological scores, clinical variables, ApoE genotype, CSF biomarkers (Aβ42, *t*-Tau and *p*-Tau) in both groups. Conversion was ascertained during follow-up. Results: EOMCI showed a longer duration of symptoms prior to the first evaluation (EOMCI = 4.57 *vs.* LOMCI = 3.31, *p* = 0.008) and scored higher on the subjective memory complaints scale (9.91 *vs.* 7.85, *p* = 0.008), but performed better in brief cognitive tests (27.81 *vs.* 26.51, *p* < 0.001 in Mini-Mental State Examination; 19.84 *vs.* 18.67, *p* = 0.005 in Montreal Cognitive Assessment) than LOMCI. ApoE genotype distribution and CSF biomarker profile were similar in both groups, as was the conversion risk. Lower Aβ42 (Hazard ratio (HR): 0.998, 95% Confidence Interval (CI) = [0.996–1.000], *p* = 0.042), higher *t*-Tau levels (HR: 1.003, 95%CI = [1.000–1.005], *p* = 0.039) and higher scores in the Alzheimer Disease Assessment Scale-Cognitive (HR: 1.186, 95%CI = [1.083–1.299], *p* = 0.002) increased the risk of conversion. Discussion: Despite differences in memory performance and memory complaints, EOMCI and LOMCI seem to represent indistinct biological groups that do not have a higher risk of conversion to AD or differ in risk factors for conversion.

## 1. Introduction

Alzheimer’s disease (AD) is the most common cause of neurodegenerative dementia [1]. Despite its higher prevalence in the older population, it is still the most frequent form of dementia under the age of 65 [2]. 

Actually, “65 years old” is an arbitrary cut-off, with no true biological meaning [1,2,3], but it is still fully considered in the most recent diagnostic criteria for AD and in selection for clinical trials. According to this conventional limit, 1%–6% of all AD patients have an early-onset disease [4]. Despite having the same histopathological characteristics, early-onset AD and late-onset AD seem to show some distinct clinical and neuropsychological profiles [5]. Research has shown a higher prevalence of non-memory symptoms (e.g., language impairment) as the initial presentation in the early-onset AD subgroup [6], as well as a faster progression of cognitive and functional decline in this early-form [7,8]. 

The pathophysiological process of AD is thought to begin many years before its clinical diagnosis [3]. The term Mild Cognitive Impairment (MCI) is used to describe subjects with demonstrable cognitive impairment who have not crossed the functional threshold for dementia. These patients have an increased risk of developing dementia, especially AD [9,10,11] and this is regarded as a prodromal stage of AD [12]. Studies conducted in reference memory clinics have shown that patients with MCI progress to AD at a rate of 10% to 15% per year [11,12,13] and 80% of these patients will have converted to AD after approximately six years of follow-up [11]. MCI was mostly described in individuals older than 65 years old, and, in fact, age has been considered a predictor of progression to dementia [14]. Apart from age, the severity of cognitive impairment [15], the ε4 allele of the apolipoprotein E (ApoE) gene [14], hippocampal atrophy [15] and a cerebrospinal fluid (CSF) biomarkers’ profile compatible with AD (*i.e.*, low CSF Aβ42 and high CSF Tau) [15], have also been reported as predictors of progression to AD.

Early-onset MCI (EOMCI) is a less characterized group and probably more heterogeneous, including more patients with other comorbidities, namely psychopathology [16,17]. Moreover, the relatively lower prevalence of AD under 65 years of age and the higher prevalence of other types of dementia [18] leads to the supposition that this heterogeneity extends to EOMCI [19]. Some evidence suggests that the impact of the risk factors for progression to dementia varies with age [20], namely ε4 allele of the ApoE [21,22] and depression [17]. 

We aimed to study MCI probably due to AD and to better characterize the clinical, genetic, and CSF biomarkers profiles and conversion rates of EOMCI, compared to the most frequent late-onset form (LOMCI). Such evidence may be relevant to an earlier and more accurate diagnosis of MCI patients and to identify risk factors for conversion to AD. 

## 2. Materials and Methods

### 2.1. Participants and Procedures

Patients were collected consecutively from June 2007 until March 2013, at the Dementia Clinic of the University Hospital of Coimbra - Portugal and in a private Memory Clinic in the same city. All patients have undergone longitudinal assessment in an annual-based comprehensive assessment (clinical and neuropsychological).

Patients with MCI probably due to AD were initially diagnosed based on the criteria purposed by the National Institute on Aging and Alzheimer’s Association Workgroup for the diagnosis of MCI due to Alzheimer’s 2011 criteria [23] as follows: (1) a subjective complaint of memory decline (reported by the subject or an informant); (2) an objective memory impairment; (3) largely normal daily life activities; and (4) absence of dementia. In order to support a clinical and cognitive syndrome consistent with that associated with the pre-dementia phase of AD, each patient had a structured clinical interview, physical and neurological examination, and a comprehensive and holistic cognitive–functional–psychological assessment battery (see Materials and neuropsychological testing). Moreover, to determine the probable degenerative cause of MCI and rule out other systemic or brain diseases, we carried out a battery of laboratory ancillary exams including complete blood count, chemistry profile, thyroid function, vitamin B12 and folic acid, ApoE genotyping, structural (computed tomography (CT) or Magnetic Resonance Imaging (MRI)) and/or functional (single-photon emission computed tomography or positron emission tomography) imaging and, eventually, CSF analysis by lumbar puncture. 

The available information was used by a multidisciplinary team to reach a consensus diagnosis and 159 patients were included. MCI-patients were further attributed to one of the two different age-of-onset groups, using the conventional division line of the 65 years old of reported onset, thus creating the EOMCI group (52 patients) and the LOMCI group (107 patients). Age at onset was estimated from subject and caregiver information using a standard questionnaire. Disease duration was established in years, from the estimated age at onset until the date of the first neuropsychological assessment [24]. Information related to family history was also taken from patients and relatives.

Follow-up time was considered until the last clinical and neuropsychological evaluation performed (for non-converters) or until conversion (for converters). Conversion to dementia was diagnosed according to the Diagnostic and Statistical Manual of Mental Disorders–IV Text Revision (DSM-IV-TR) [25] and, specifically for AD, using the most recent National Institute of Neurological and Communicative Diseases and Stroke/Alzheimer’s Disease and Related Disorders Association (NINCDS-ADRDA) criteria [26]. The final diagnosis of dementia was confirmed by the coordinator of this study, based in the annual-based comprehensive assessment.

As exclusion criteria for enrollment, we considered a significant underlying medical or neurological illness that could account for the decline in cognition revealed by lab tests or imaging; a relevant psychiatric disease, including major depression, suggested in the medical interview and confirmed by specific scales; CT or MRI demonstration of significant vascular burden [27] (large cortico-subcortical infarct; extensive subcortical white matter lesions superior to 25%; uni- or bilateral thalamic lacune; lacune in the head of the caudate nucleus; and more than 2 lacunes).

The present research complied with the ethical guidelines for human experimentation stated in the Declaration of Helsinki and was approved by the Ethics Board of Coimbra University Hospital. An informed oral consent was obtained from all the participants after the aims and procedures of the investigation were fully explained by a member of the study group. 

### 2.2. Material and Neuropsychological Testing

At baseline, all participants underwent a comprehensive and holistic cognitive–functional–psychological standard assessment, carried out by the same team of trained neuropsychologists, comprising several tests and scales adapted and validated to Portuguese population: (1) Cognitive instruments, including the Mini-Mental State Examination (MMSE) [28,29], the Montreal Cognitive Assessment (MoCA) [30,31], the Alzheimer Disease Assessment Scale-Cognitive (ADAS-Cog) [32,33] and a comprehensive neuropsychological battery (Battery of Lisbon for the Assessment of Dementia [34]), used for diagnostic purposes (*data not shown*). (2) The Blessed Dementia Scale [35] and the Clinical Dementia Rating (CDR) [36,37] were used for global staging. Considering this last scale, CDR = 0 indicates no impairment and CDR = 0.5, 1, 2 and 3 indicate questionable, mild, moderate and severe dementia, respectively. We also used the CDR-sum of boxes quantitative score, obtained by adding the result of each box. (3) The Disability Assessment for Dementia (DAD) [38] scale and the Activities of Daily Living (ADL) [39] was also used to assess functional performance. (4) The Geriatric Depression Scale (GDS [40,41]) was applied to exclude major depression. (5) The Neuropsychiatric Inventory (NPI) [42] was used to evaluate concomitant psychiatric disorders. (6) The Subjective Memory Complaints (SMC) [43] questionnaire was applied to the patient and the caregiver—both scores were used for comparison of complaints (regarding the patient’s deficits) and identification of true impairment. 

The available information was used to reach the diagnostic criteria for MCI due to Alzheimer’s disease [26]: the core objective memory deficit was considered when scores on standard Wechsler memory tests [44] (included in the Battery of Lisbon for the Assessment of Dementia) were >1.5 standard deviations below age/education adjusted norms (with or without deficits in other cognitive domains); information related to preservation of independence in functional abilities was enhanced by the results of functional scales (DAD and CDR). Conversion to Frontotemporal dementia (FTD) was considered according to the 2011 revised criteria for the behavioral variant of frontotemporal dementia [45].

As stated previously, we performed an annual-based comprehensive assessment with the referred scales to detect objective evidence of progressive decline over time and for establishing a conversion to AD according to clinical diagnostic criteria for probable AD [25,26,27,28,29,30,31,32,33,34,35,36,37,38,39,40,41,42,43,44,45,46].

### 2.3. Genotyping and CSF biomarkers

For ApoE genotyping, DNA was isolated from whole EDTA-blood using a commercial kit (Roche Diagnostics GmbH, Manheim, Germany) and ApoE genotype was determined by polymerase chain reaction-restriction fragment length polymorphisms (PCR-RFLP) assay, as previously described [47].

CSF samples were collected from a subgroup of patients as part of their routine clinical diagnosis investigation. Pre-analytical and analytical procedures were done in accordance with the Alzheimer’s Association guidelines for CSF biomarker determination [48]. Briefly, CSF samples were collected in sterile polypropylene tubes, immediately centrifuged at 1800 g for 10 min at 4 °C, aliquoted into polypropylene tubes and stored at −80 °C until analysis. CSF Aβ42, total Tau (*t*-Tau) and phosphorylated Tau (*p*-Tau) were measured separately by commercially available sandwich ELISA kits (Innotest, Innogenetics, Ghent, Belgium), as previously described [49,50]. External quality control of the assays was performed under the scope of the Alzheimer’s Association Quality Control Program for CSF Biomarkers [48]. A CSF profile typical of AD was defined as a score below 1 calculated with the formula Aβ42/[240 + (1.18 × Tau)] [51]. This formula has been shown to distinguish patients with AD from controls or from patients with other types of dementia and can identify patients with prodromal AD amongst MCI cases [52,53].

### 2.4. Statistical Analysis

Statistical analysis was performed using Statistical Package for the Social Sciences (SPSS) version 20 for windows. Parametric analyses were performed, considering samples greater than 30 as with normal distribution. Independent-samples t tests were performed to compare demographic data, SMC (both patient and caregiver independent scores), CDR, DAD, ADL, GDS, NPI and CSF biomarkers. ANCOVA was used to compare MMSE, MoCA, ADAS-cog and Blessed scores, all adjusted to education and age. Non-parametric analysis (χ-square test) was performed for the presence of ApoE ε4 alleles. All the parameters that showed statistical significant differences between groups were entered in a Cox regression model to study conversion to AD. Well-established risk factors, such as gender, years of education, positive family history, ε4 allele and CSF biomarkers were also considered in the model. Age at onset was not considered as it is represented by late and early onset subsets. MoCA and MMSE scores were excluded for being co-dependent with ADAS-cog scores and education. Statistical significance was set at *p* < 0.05.

## 3. Results

The study sample consisted of 159 patients: 52 with EOMCI and 107 with LOMCI. The characterization of the study sample and details of both subgroups is provided in Table 1 (sample size, age-at-onset, education level, gender, family history, apoE genotype, disease duration, time of follow-up and percentage of converters). Groups were matched for education (*p* = 0.237) and gender (*p* = 0.715) and there were no significant differences for positive family history of dementia (*p* = 0.344) that was positive in approximately half of the patients. The average time of follow-up was 23.63 months (±25.60), with no significant differences between groups (*p* = 0.962). There was also no difference between groups in the percentage of ε4 allele carriers, which reached 43% of the overall study population (*p* = 0.969; data available in 148 of the 159 patients). 

As anticipated, there were statistically significant differences in terms of age at first evaluation and at onset of complaints (*p* < 0.001), but the duration of symptoms previously to the first evaluation was larger for EOMCI patients (EOMCI = 4.57 ± 2.90 years; LOMCI = 3.31 ± 2.45 years; *p* = 0.008). Regarding clinical conversion at follow-up, 38.1% of MCI patients converted to dementia (59 out of 155 patients with available follow-up information). Although the LOMCI group had a slightly increased percentage of converters (41.8% *vs.* 31.4%), this difference did not reach statistical significance (*p* = 0.358). Of the fifty-nine converters, 57 converted to AD and the other two to FTD.

The comparisons made between neuropsychological tests are presented in Table 2. Statistically significant differences were found in MMSE (EOMCI = 27.81 ± 2.48; LOMCI = 26.51 ± 2.69, *p* < 0.001), MoCA (EOMCI = 19.84 ± 5.04; LOMCI = 18.67 ± 4.57, *p* = 0.005) and ADAS-cog (EOMCI = 9.17 ± 5.16; LOMCI = 11.12 ± 5.04, *p* < 0.001), with EOMCI subjects showing an overall better performance. The EOMCI group also presented a significantly higher SMC (patient score) (*p* = 0.008). This difference was not observed when the same questionnaire was administered to the caregivers (*p* = 0.773). The groups showed similar performance in all the other applied scales with no statistical significant difference (Table 2). Noteworthy, NPI scores in the LOMCI group were higher than in the EOMCI, showing a tendency for statistical significance.

CSF samples were available for 76 patients (34 EOMCI and 42 LOMCI), and the results of the core CSF biomarkers Aβ42, *t*-Tau and *p*-Tau are presented in Table 3. There were no differences between the subgroups for the mean levels of Aβ42 (*p* = 0.456), *t*-Tau (*p* = 0.773) and *p*-Tau (*p* = 0.594). Fifty-one out of the 76 MCI patients (67.1%) presented a CSF profile compatible with AD, indicative of a high risk of conversion. Although this percentage was larger in LOMCI (73.8%) than in EOMCI patients (58.8%), the difference was not statistically significant (*p* = 0.167).

**Figure 1:**
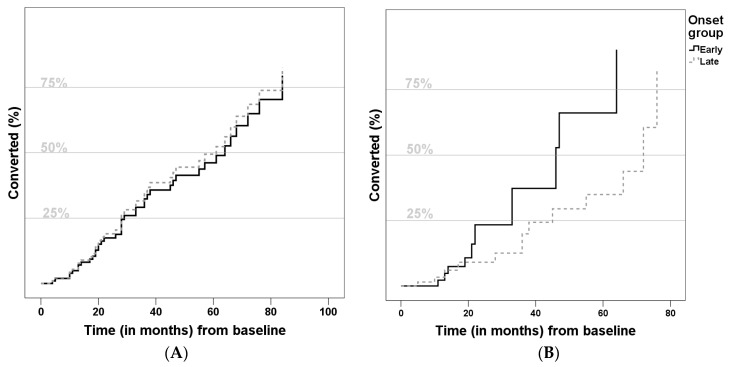
Comparison of conversion to Alzheimer disease curves (**A**) *not adjusted*; and (**B**) adjusted for gender, years of education, previous disease duration, family history, ApoE-ε4 allele, Cerebrospinal fluid biomarkers and Alzheimer Disease Assessment Scale) of Late-Onset Mild Cognitive Impairment (LOMCI) (**A**) Hazard Ratio: 0.722, 95%Confidence Interval = [0.418, 1.247], *p* = 0.243; (**B**) Hazard Ratio: 0.721, 95%Confidence Interval = [0.264, 1.970], *p* = 0.524) and Early-Onset Mild Cognitive Impairment (EOMCI).

Relative conversion risk to AD was determined using a Cox regression model (Figure 1). As presented, the groups did not differ with statistical significance, but the LOMCI group showed a lower risk of conversion (Hazard ratio (HR): 0.721, 95%Confidence Interval (CI) = [0.264, 1.970], *p* = 0.524). Considering predictors of conversion to AD, lower Aβ42 (HR: 0.998, 95%CI = [0.996,1.000], *p* = 0.042), higher *t*-Tau (HR: 1.003, 95%CI = [1.000, 1.005], *p* = 0.039) and higher ADAS-Cog score (HR: 1.186, 95%CI = [1.083, 1.299], *p* = 0.002) increased the risk of conversion. None of the other variables (gender, years of education, years of previous disease duration, positive family history, apoE4 or *p*-Tau) were significant predictors.

## 4. Discussion

Previous studies comparing EOMCI and LOMCI are scarce [8,9,10,11,12,13,14,15,16,17,18,19,20,21,22,23,24,25,26,27,28,29,30,31,32,33,34,35,36,37,38,39,40,41,42,43,44,45,46,47,48,49,50,51,52,53,54] and failed to show individual group differences more than MCI characteristics and conversion predictors as a whole. Kim and colleagues [54] followed 28 patients for five years, with special attention to PET and neuropsychological data. In their study, EOMCI patients had better performances only in verbal recall and word-fluency tests, even though they showed more extensive hypometabolism on FDG-PET than LOMCI patients. Ye and colleagues followed 425 patients with amnestic-MCI for around 1.5 years [8], investigating neuropsychological characteristics and risk factors for conversion. They showed that EOMCI patients with visuo-spatial memory impairment and LOMCI patients with poor verbal memory were in higher risk for conversion.

In our study, as expected and designed, the groups were clearly age distinct, with a mean difference around 13 years, an essential premise for the proposed analysis. They were equivalent in all other demographic data, as there were no significant differences in terms of gender, education and family history of dementia. However, they were different in terms of duration of symptoms previously to the first evaluation, with the EOMCI patients having an unexpected larger delay between onset of complaints and arrival at our outpatient clinic. This has already been reported in dementia settings [55,56], but not in MCI cases, and may be related to referral biases, namely a lower suspicion or higher disbelief of true cognitive decline in younger patients. 

In fact, unawareness of memory impairment may interfere in the investigation and referral process. In order to explore the awareness of the impairment, we applied and compared the SMC scale both to the patient and to the caregiver. When caregivers answered the questions about the patients, no differences arose, implying that both groups would be similarly impaired. However, when the same questions were applied to the patients, the EOMCI groups had significantly higher scores, implying that those patients might have more insight about their condition. Similar results have been described in AD patients, in which anosognosia was associated with advanced age, lower education level and more marked behavioral symptoms [57]. This result suggests that there could be other reasons other than lack of awareness to the opposite higher lag between onset and evaluation, namely fear of the diagnosis, lower decline rate or higher diagnostic disbelief. 

Considering the cognitive rating scales adjusted to the level of education, results showed significant differences (MMSE and ADAS-cog, *p* < 0.001; MoCA, *p* = 0.005) with EOMCI subjects presenting a better general cognitive level. The same trend for better performance in EOMCI was observed in previous studies [8,9,10,11,12,13,14,15,16,17,18,19,20,21,22,23,24,25,26,27,28,29,30,31,32,33,34,35,36,37,38,39,40,41,42,43,44,45,46,47,48,49,50,51,52,53,54] and also in dementia [58]. This seems to indicate that these patients are either diagnosed in an earlier phase of the disease or, more likely (according to all results), that they have a higher cognitive reserve which may act as a buffer for cognitive decline. This also might be the reason why EOMCI have a larger delay from onset of the symptoms and evaluation at a reference clinic.

In our cohort, the equivalence between groups in GDS and NPI scores suggests that both psychopathology, in terms of prevalence or severity, are not strongly distinguishing factors of these age groups. Neuropsychiatric symptoms, namely depression, are considered a risk factor for MCI [59] and AD [60,61,62], namely in older patients [17]. This trend is observable in our cohort, although without statistical significance. In addition, depressive symptoms are associated with overdiagnosis of MCI, which tends to occur in younger patients [63]. However, we should take into account that this may suffer from a referral bias, as patients with higher neuropsychiatric burden may tend to be referred for the psychiatry outpatient clinic rather than to general neurology or dementia clinic. 

The use of CSF biomarkers can improve the identification of AD pathology in patients with MCI and predict the likelihood of progression to dementia, especially within a relatively short period [64,65]. The altered pattern of CSF protein levels usually seen in patients with AD is also present in MCI (especially in patients who will convert to AD): high levels of *t*-Tau and/or *p*-Tau and decreased Aβ42 levels [65]. In our sample, no significant differences were found between groups regarding the mean levels of these biomarkers. Aβ42 levels were slightly higher in the EOMCI group than in the LOMCI. Also, *t*-Tau and *p*-Tau levels seemed to be lower, which could be attributed to age [66]. Moreover, CSF profiles were also comparable between groups, although suggesting a higher prevalence of AD-compatible CSF in LOMCI. This could be explained by the mixed-etiology of the impairments that are thought to be included in the EOMCI group [18,19]. The percentage of MCI patients that we identified as having a CSF AD profile (67%) is somewhat higher than the one reported by other studies [65,67,68]. Moreover, this percentage is in line with the results of our Cox regression model, that estimates a rate of conversion from MCI to dementia of 70% in 6 years, and also with previous studies [69].

Overall, the conversion rate of our cohort (around 11.6%/year) is convergent with the literature [11,70]. Besides, in our sample, the relative risk of conversion was similar in both groups (Figure 1), which agrees with the only known study which addressed this particular question [8]. We also confirmed some statistically significant risk factors for conversion, such as higher ADAS-cog score (indicating worse cognitive performance), lower Aβ42 and higher Tau levels. All of these are known risk factors for conversion to AD [15,64,65,71].

This study has some limitations, namely in the sample size: we have two groups with different sizes and variable time of follow-up. Secondly, this study did not consider MRI data, namely measures of atrophy and microvascular burden, which would give further information, namely the degree to which the probable higher microvascular burden in LOMCI contributed to the worsening of cognitive performance in that group, as described in other studies [72,73]. This might be addressed in forthcoming studies. There may be a referral biases, namely of those patients presenting with psychiatric or more bizarre changes, whom may be preferentially referred for psychiatric evaluation. We also excluded those with major depression, as this would explain cognitive impairment. However, there is the possibility that some of these patients are suffering from a true neurodegenerative process. As most of these patients were followed for several months and the diagnosis is revised in every appointment, this error may be softened. Finally, histopathological confirmation was not performed, so we cannot exclude other pathological processes interfering with the cognitive decline. On the other hand, our study makes up for some of these limitations by comprising ApoE genotyping of almost the entire sample and CSF biomarkers for about half of the sample, which have strong influence on the evolution of the cognitive decline. Also, our sample was followed-up for an average of three years and we have an extensive and comprehensive evaluation, including subjective memory complaints, neuropsychological, neuropsychiatric and functional status formal assessments.

## 5. Conclusions

In the end, there was no difference in terms of conversion between groups. EOMCI and LOMCI did not differ in terms of CSF biomarkers or psychiatric comorbidities. Despite having more memory complaints, EOMCI patients took longer to be evaluated in a tertiary center and showed a better initial general performance, which may be explained by the conjunction of a higher cognitive reserve and diagnostic disbelief. Overall, higher Tau, lower Aβ42 and higher ADAS-cog were risk factors for conversion to AD, independently of the age group.

## Figures and Tables

**Table 1:** Demographic, clinical and genetic variables in the studied population.

	EOMCI	LOMCI	Total	*p*
n	52	107	159	
Age at onset	60.0 (±4.99)	73.2 (±5.31)	68.5 (±8.15)	<0.001
Age at first evaluation	64.7 (±5.57)	76.1 (±5.39)	72.4 (±7.65)	<0.001
Education (years)	5.62 (±3.09)	6.35 (±4.58)	5.88 (±4.01)	0.298
Gender (female %)	59.6	62.6	61.6	0.715
Positive family history (%)	53.3	44.8	47.5	0.344
ApoE-ε4 carriers (%)	43.5	43.1	43.2	0.969
Disease duration at first evaluation (years)	4.57 (±2.90)	3.31 (±2.45)	3.76 (±2.69)	0.008
Time of follow-up (months)	38.54 (±30.23)	37.44 (±24.46)	40.03 (±26.90)	0.814
Converters (%)	31.4	41.8	38.3	0.358

Results are displayed either as mean (±standard deviation) or percentage of the referred variable in the considered sample. EOMCI: Early-onset Mild Cognitive Impairment; LOMCI: Late-onset Mild Cognitive Impairment.

**Table 2:** Comparison of neuropsychological tests scores between groups.

	EOMCI Mean (±SD)	LOMCI Mean (±SD)	*p*
SMC-patient	9.91 (±3.29)	7.85 (±3.85)	0.008
SMC-caregiver	8.58 (±3.62)	8.32 (±3.69)	0.773
MMSE *	27.81 (±2.48)	26.51(±2.69)	<0.001
MoCA *	19.84 (±5.04)	18.67 (±4.57)	0.003
ADAS-cog *	9.17 (±5.16)	11.12 (±5.04)	<0.001
Blessed *	1.63 (±1.52)	2.29 (±2.46)	0.707
CDR-sob	1.41 (±0.97)	1.577 (±1.01)	0.332
CDR-memory	0.56 (±0.17)	0.60 (±0.20)	0.287
CDR-orientation	0.21 (±0.29)	0.30 (±0.34)	0.100
CDR-judgement and problem-solving	0.32 (±0.30)	0.33 (±0.31)	0.746
CDR-activity	0.13 (±0.22)	0.18 (±0.27)	0.279
CDR-home and hobbies	0.12 (±0.24)	0.16 (±0.26)	0.321
CDR-personal care	0.00 (±0.00)	0.02 (±0.14)	0.158
DAD	95.12 (±5.26)	93.92 (±6.18)	0.391
ADL	1.57 (±1.55)	1.446 (±1.79)	0.819
GDS	12.21 (±5.75)	10.33 (±6.84)	0.200
NPI	4.56 (±5.72)	7.44 (±9.06)	0.058

Mean scores ±standard deviation) are presented for each group. * marks variables where the comparison test was adjusted for education and age. EOMCI: Early-onset Mild Cognitive Impairment; LOMCI: Late-onset Mild Cognitive Impairment; SMC: Subjective Memory Complaints; MMSE: Mini-Mental State Examination; MoCA: Montreal Cognitive Assessment; ADAS-Cog: Alzheimer Disease Assessment Scale-Cognitive; CDR: Clinical Dementia Rating; CDR-sob: CDR-sum of boxes; DAD: Disability Assessment for Dementia; ADL: Activities of Daily Living; GDS: Geriatric Depression Scale; NPI: Neuropsychiatric Inventory. Note: In MMSE, MoCA, DAD and ADL lower scores are indicative of worse performance. In SMC, ADAS-cog, Blessed, CDR, GDS and NPI higher scores, are indicative of worse performance or higher impairment.

**Table 3:** Characterization of CSF biomarkers in the study population.

	EOMCI (n = 34)	LOMCI (n = 42)	*p*
Aβ42 (pg/mL)	649.7 (±339.1)	591.3 (±336.7)	0.456
*t*-Tau (pg/mL)	437.2 (±396.0)	459.3 (±227.1)	0.773
*p*-Tau (pg/mL)	57.1 (±38.2)	61.3 (±29.7)	0.594
CSF-AD profile (%)	58.8	73.8	0.167

CSF-AD profile was defined as a score below 1 with the formula Aβ42/[240 + (1.18 × Tau). EOMCI: Early-onset Mild Cognitive Impairment; LOMCI: Late-onset Mild Cognitive Impairment. AD: Alzheimer’s Dementia.

## References

[1] Lobo A., Launer L.J., Fratiglioni L., Andersen K., Di Carlo A., Breteler M.M., Copeland J.R., Dartigues J.F., Jagger C., Martinez-Lage J. (2000). Prevalence of dementia and major subtypes in Europe: A collaborative study of population-based cohorts. Neurolo. Dise. Elder. Res. Group Neurol..

[2] Harvey R.J., Skelton-Robinson M., Rossor M.N. (2003). The prevalence and causes of dementia in people under the age of 65 years. J. Neurol. Psychiatry.

[3] Morris J.C. (2005). Early-stage and preclinical Alzheimer disease. Alzheimer Dis. Assoc. Disord..

[4] Bird T.D., Pagon R.A., Adam M.P., Bird T.D., Dolan C.R., Fong C.T., Stephens K. (1993). Alzheimer Disease Overview. GeneReviews^®^.

[5] Sa F., Pinto P., Cunha C., Lemos R., Letra L., Simoes M., Santana I. (2012). Differences between Early and Late-Onset Alzheimer’s Disease in Neuropsychological. Tests. Front. Neurol..

[6] Licht E.A., McMurtray A.M., Saul R.E., Mendez M.F. (2007). Cognitive differences between early- and late-onset. Am. J. Alzheimer’s Dis. Dement..

[7] Wilson R.S., Li Y., Aggarwal N.T., Barnes L.L., McCann J.J., Gilley D.W., Evans D.A. (2004). Education and the course of cognitive decline in Alzheimer disease. Neurology.

[8] Ye B.S., Seo S.W., Lee Y., Kim S.Y., Choi S.H., Lee Y.M., Kim H., Han H.J., Na D.L., Kim E.J. (2012). Neuropsychological performance and conversion to Alzheimer’s disease in early- compared to late-onset amnestic mild cognitive impairment: CREDOS study. Dement. Geriatric. Cogn. Disord..

[9] Bowen J., Teri L., Kukull W., McCormick W., McCurry S.M., Larson E.B. (1997). Progression to dementia in patients with isolated memory loss. Lancet.

[10] Morris J.C., Cummings J. (2005). Mild cognitive impairment (MCI) represents early-stage Alzheimer’s disease. J. Alzheimer’s Dis..

[11] Petersen R.C., Smith G.E., Waring S.C., Ivnik R.J., Tangalos E.G., Kokmen E. (1999). Mild cognitive impairment: Clinical characterization and outcome. Arch. Neurol..

[12] Dubois B., Feldman H.H., Jacova C., Cummings J.L., DeKosky S.T., Barberger-Gateau P., Delacourte A., Frisoni F., Fox N.C., Galasko D. (2010). Revising the definition of Alzheimer’s disease: A new lexicon. Lancet Neurol..

[13] Tierney M.C., Szalai J.P., Dunn E., Geslani D., McDowell I. (2000). Prediction of probable Alzheimer disease in patients with symptoms suggestive of memory impairment: Value of the Mini-Mental State Examination. Arch. Fam. Med..

[14] Petersen R.C., Smith G.E., Ivnik R.J., Tangalos E.G., Schaid D.J., Thibodeau S.N., Kokmen E., Waring S.C., Kurland L.T. (1995). Apolipoprotein E status as a predictor of the development of Alzheimer’s disease in memory-impaired individuals. J. Am. Med. Assoc..

[15] Risacher S.L., Saykin A.J., West J.D., Shen L., Firpi H.A., McDonald B. (2009). Baseline MRI predictors of conversion from MCI to probable AD in the ADNI cohort. Curr. Alzheimer Res..

[16] Apostolova L.G., Di L.J., Duffy E.L., Brook J., Elashoff D., Tseng C.H., Fairbanks L., Cummings J.L. (2014). Risk factors for behavioral abnormalities in mild cognitive impairment and mild Alzheimer’s disease. Dement. Geriatr. Cogn. Disord..

[17] Sachs-Ericsson N., Moxley J.H., Corsentino E., Rushing N.C., Sheffler J., Selby E.A., Gotlib I., Steffens D.C. (2014). Melancholia in later life: Late and early onset differences in presentation, course, and dementia risk. Int. J. Geriatr. Psychiatry.

[18] Rossor M.N., Fox N.C., Mummery C.J., Schott J.M., Warren J.D. (2010). The diagnosis of young-onset dementia. Lancet Neurol..

[19] Anstey K.J., Cherbuin N., Eramudugolla R., Sargent-Cox K., Easteal S., Kumar R., Sachdev P. (2013). Characterizing mild cognitive disorders in the young-old over 8 years: Prevalence, estimated incidence, stability of diagnosis, and impact on IADLs. Alzheimer’s Demen..

[20] Ganguli M., Lee C.W., Snitz B.E., Hughes T.F., McDade E., Chang C.C. (2015). Rates and risk factors for progression to incident dementia vary by age in a population cohort. Neurology.

[21] Rusted J.M., Evans S.L., King S.L., Dowell N., Tabet N., Tofts P.S. (2013). APOE e4 polymorphism in young adults is associated with improved attention and indexed by distinct neural signatures. NeuroImage.

[22] Marchant N.L., King S.L., Tabet N., Rusted J.M. (2010). Positive effects of cholinergic stimulation favor young APOE epsilon4 carriers. Neuropsychopharmacology.

[23] Albert M.S., DeKosky S.T., Dickson D., Dubois B., Feldman H.H., Fox N.C., Gamst A., Holtzman D.M., Jagust W.J., Petersen R.C. (2011). The diagnosis of mild cognitive impairment due to Alzheimer’s disease: Recommendations from the National Institute on Aging-Alzheimer’s Association workgroups on diagnostic guidelines for Alzheimer’s disease. Alzheimer’s Dement..

[24] Sano M., Devanand D.P., Richards M., Miller L.W., Marder K., Bell K., Dooneief G., Bylsma F.W., Lafleche G., Albert M. (1995). A standardized technique for establishing onset and duration of symptoms of Alzheimer’s disease. Archives Neurol..

[25] (2013). Diagnostic and Statistical Manual of Mental Disorders.

[26] McKhann G.M., Knopman D.S., Chertkow H., Hyman B.T., Jack C.R., Kawas C.H., Klunk W.E., Koroshetz W.J., Manly J.J., Mayeux R. (2011). The diagnosis of dementia due to Alzheimer’s disease: Recommendations from the National Institute on Aging-Alzheimer’s Association workgroups on diagnostic guidelines for Alzheimer’s disease. Alzheimer’s Dement..

[27] Roman G.C., Tatemichi T.K., Erkinjuntti T., Cummings J.L., Masdeu J.C., Garcia J.H., Amaducci L., Orgogozo J.-M., Brun A., Hofman A. (1993). Vascular dementia: Diagnostic criteria for research studies. Report of the NINDS-AIREN International Workshop. Neurology.

[28] Guerreiro M.S., Botelho P., Leitao M., Adaptaçao O. (1994). A populaçao portuguesa da traduçao do "Mini Mental State Examination" (MMSE). Rev. Port. Neurologia.

[29] Folstein M.F., Folstein S.E., McHugh P.R. (1975). “Mini-mental state”: A practical method for grading the cognitive state of patients for the clinician. J. Psychiatric Res..

[30] Freitas S., Simoes M.R., Alves L., Santana I. (2011). Montreal Cognitive Assessment (MoCA): Normative study for the Portuguese population. J. Clin. Exp. Neuropsychol..

[31] Nasreddine Z.S., Phillips N.A., Bedirian V., Charbonneau S., Whitehead V., Collin I., Cummings J.L., Chertkow H. (2005). The Montreal Cognitive Assessment, MoCA: A brief screening tool for mild cognitive impairment. J. Am. Geriatrics Soc..

[32] Guerreiro M., Fonseca S., Barreto J., Garcia C. (2008). Escalas e Testes na Demencia. GEECD.

[33] Mohs R.C., Rosen W.G., Davis K.L. (1983). The Alzheimer’s disease assessment scale: an instrument for assessing treatment efficacy. Psychopharmacol. Bull..

[34] Guerreiro M. (2008). Contributo da Neuropsicologia Para o Estudo Das Demencias [Contribution of Neuropsychology to the study of dementia].

[35] Garcia C., Silva G.M., Botelho A.P., Leitão M.A., Castro A., Caldas A. (2008). Avaliação Breve do Estado Mental: Escalas e Testes na Demência. GEECD.

[36] Morris J.C. (1993). The Clinical Dementia Rating (CDR): Current version and scoring rules. Neurology.

[37] Garrett C., Santos F., Tracana I., Barreto J., Sobral M., Fonseca R. (2008). Avaliação Clínica da Demência. Escalas e Testes na Demência. GEECD.

[38] Leitão O. (2008). Escalas e Testes na Demência. GEECD.

[39] Lino V.T., Pereira S.R., Camacho L.A., Ribeiro Filho S.T., Buksman S. (2008). Cross-cultural adaptation of the Independence in Activities of Daily Living Index (Katz Index). Cad Saude Publ..

[40] Yesavage J.A., Brink T.L., Rose T.L., Lum O., Huang V., Adey M., Leirer V.O. (1982). Development and validation of a geriatric depression screening scale: A preliminary report. J. Psychiatric Res..

[41] Barreto J.L.A., Santos F., Sobral M. (2008). Escala de Depressão Geriátrica [Geriatric Depression Scale] Escalas e Testes na Demência. GEECD.

[42] Leitão O., Nina A. (2008). Escalas e Testes na Demência. GEECD.

[43] Mendes T., Gino S., Ribeiro F., Guerreiro M., de Sousa G., Ritchie K., de Mendonca A. (2008). Memory complaints in healthy young and elderly adults: Reliability of memory reporting. Aging Ment. Health.

[44] (2008). Wechsler. WMS-III: Escala de Memória de Wechsler.

[45] Rascovsky K., Hodges J.R., Knopman D., Mendez M.F., Kramer J.H., Neuhaus J., van Swieten J.C., Seelaar H., Dopper E.G., Onyike C.U. (2011). Sensitivity of revised diagnostic criteria for the behavioural variant of frontotemporal dementia. Brain.

[46] McKhann G., Drachman D., Folstein M., Katzman R., Price D., Stadlan E.M. (1984). Clinical diagnosis of Alzheimer’s disease: Report of the NINCDS-ADRDA Work Group under the auspices of Department of Health and Human Services Task Force on Alzheimer’s Disease. Neurology.

[47] Crook R., Hardy J., Duff K. (1994). Single-day apolipoprotein E genotyping. J. Neurosci. Methods.

[48] Mattsson N., Andreasson U., Persson S., Arai H., Batish S.D., Bernardini S., Bocchio-Chiavetto L., Blankenstein M.A., Carrillo M.C., Chalbot S. (2011). The Alzheimer’s Association external quality control program for cerebrospinal fluid biomarkers. Alzheimer’s Dement..

[49] Baldeiras I.E., Ribeiro M.H., Pacheco P., Machado A., Santana I., Cunha L., Oliveira C.R. (2009). Diagnostic value of CSF protein profile in a Portuguese population of sCJD patients. J. Neurol..

[50] Kapaki E., Kilidireas K., Paraskevas G.P., Michalopoulou M., Patsouris E. (2001). Highly increased CSF Tau protein and decreased beta-amyloid (1-42) in sporadic CJD: A discrimination from Alzheimer’s disease?. J. Neurol. Neurosurg. Psychiatry.

[51] Hulstaert F., Blennow K., Ivanoiu A., Schoonderwaldt H.C., Riemenschneider M., De Deyn P.P., Bancher C., Cras P., Wiltfang J., Mehta P.D. (1999). Improved discrimination of AD patients using beta-amyloid(1-42) and tau levels in CSF. Neurology.

[52] Andreasen N., Minthon L., Vanmechelen E., Vanderstichele H., Davidsson P., Winblad B., Blennow K. (1999). Cerebrospinal fluid tau and Abeta42 as predictors of development of Alzheimer’s disease in patients with mild cognitive impairment. Neurosci. Lett..

[53] Visser P.J. (2009). Use of biomarkers to select the target population for clinical trials in subjects with mild cognitive impairment. J. Nutr. Health Aging.

[54] Kim S.H., Seo S.W., Yoon D.S., Chin J., Lee B.H., Cheong H.K., Han S.H., Na D.L. (2010). Comparison of neuropsychological and FDG-PET findings between early- *versus* late-onset mild cognitive impairment: A five-year longitudinal study. Dement. Geriatr. Cogn. Disord..

[55] Papageorgiou S.G., Kontaxis T., Bonakis A., Kalfakis N., Vassilopoulos D. (2009). Frequency and causes of early-onset dementia in a tertiary referral center in Athens. Alzheimer Dis. Assoc. Disord..

[56] Shinagawa S., Ikeda M., Toyota Y., Matsumoto T., Matsumoto N., Mori T., Ishikawa T., Fukuhara R., Komori K., Hokoishi K. (2007). Frequency and clinical characteristics of early-onset dementia in consecutive patients in a memory clinic. Dement. Geriatr. Cogn. Disord..

[57] Castrillo Sanz A., Andres Calvo M., Repiso Gento I., Izquierdo Delgado E., Gutierrez Rios R., Rodriguez Herrero R., Rodriguez Sanz F., Tola-Arribas M.A. (2015). Anosognosia in Alzheimer disease: Prevalence, associated factors, and influence on disease progression. Neurologia.

[58] McMurtray A., Clark D.G., Christine D., Mendez M.F. (2006). Early-onset dementia: Frequency and causes compared to late-onset dementia. Dement. Geriatr. Cogn. Disord..

[59] Geda Y.E., Roberts R.O., Mielke M.M., Knopman D.S., Christianson T.J., Pankratz V.S., Boeve B.F., Sochor O., Tangalos E.G., Petersen R.C. (2014). Baseline neuropsychiatric symptoms and the risk of incident mild cognitive impairment: A population-based study. Am. J. Psychiatry.

[60] Gao Y., Huang C., Zhao K., Ma L., Qiu X., Zhang L., Xiu Y., Chen L., Lu W., Huang C. (2013). Depression as a risk factor for dementia and mild cognitive impairment: A meta-analysis of longitudinal studies. Int. J. Geriatr. Psychiatry.

[61] Monastero R., Mangialasche F., Camarda C., Ercolani S., Camarda R. (2009). A systematic review of neuropsychiatric symptoms in mild cognitive impairment. J. Alzheimer’s Dis..

[62] Cooper C., Sommerlad A., Lyketsos C.G., Livingston G. (2015). Modifiable predictors of dementia in mild cognitive impairment: A systematic review and meta-analysis. Am. J. Psychiatry.

[63] Edmonds E.C., Delano-Wood L., Galasko D.R., Salmon D.P., Bondi M.W. (2014). Alzheimer’s Disease Neuroimaging I. Subjective cognitive complaints contribute to misdiagnosis of mild cognitive impairment. J. Int. Neuropsychol. Soc..

[64] Jack C.R., Wiste H.J., Vemuri P., Weigand S.D., Senjem M.L., Zeng G., Bernstein M.A., Gunter J.L., Pankratz V.S., Aisen P.S. (2010). Brain beta-amyloid measures and magnetic resonance imaging atrophy both predict time-to-progression from mild cognitive impairment to Alzheimer’s disease. Brain.

[65] Hansson O., Zetterberg H., Buchhave P., Londos E., Blennow K., Minthon L. (2006). Association between CSF biomarkers and incipient Alzheimer’s disease in patients with mild cognitive impairment: A follow-up study. Lancet Neurol..

[66] Silverberg G.D., Miller M.C., Messier A.A., Majmudar S., Machan J.T., Donahue J.E., Stopa E.G., Johanson C.E. (2010). Amyloid deposition and influx transporter expression at the blood-brain barrier increase in normal aging. J. Neuropathol. Exp. Neurol..

[67] Visser P.J., Verhey F., Knol D.L., Scheltens P., Wahlund L.O., Freund-Levi Y., Tsolaki M., Minthon L., Wallin A.K., Hampel H. (2009). Prevalence and prognostic value of CSF markers of Alzheimer’s disease pathology in patients with subjective cognitive impairment or mild cognitive impairment in the DESCRIPA study: A prospective cohort study. Lancet Neurol..

[68] Buchhave P., Minthon L., Zetterberg H., Wallin A.K., Blennow K., Hansson O. (2012). Cerebrospinal fluid levels of beta-amyloid 1-42, but not of tau, are fully changed already 5 to 10 years before the onset of Alzheimer dementia. Arch. Gen. Psychiatry.

[69] Petersen R.C., Stevens J.C., Ganguli M., Tangalos E.G., Cummings J.L., DeKosky S.T. (2001). Practice parameter: Early detection of dementia: Mild cognitive impairment (an evidence-based review). Report of the Quality Standards Subcommittee of the American Academy of Neurology. Neurology.

[70] Tierney M.C., Szalai J.P., Snow W.G., Fisher R.H., Nores A., Nadon G., Dunn E., St George-Hyslop P.H. (1996). Prediction of probable Alzheimer’s disease in memory-impaired patients: A prospective longitudinal study. Neurology.

[71] Llano D.A., Laforet G., Devanarayan V. (2011). Alzheimer’s Disease Neuroimaging I. Derivation of a new ADAS-cog composite using tree-based multivariate analysis: Prediction of conversion from mild cognitive impairment to Alzheimer disease. Alzheimer Dis. Assoc. Disord..

[72] Tosto G., Zimmerman M.E., Carmichael O.T., Brickman A.M. (2014). Alzheimer’s Disease Neuroimaging I. Predicting aggressive decline in mild cognitive impairment: the importance of white matter hyperintensities. JAMA Neurol..

[73] Defrancesco M., Marksteiner J., Deisenhammer E., Kemmler G., Djurdjevic T., Schocke M. (2013). Impact of white matter lesions and cognitive deficits on conversion from mild cognitive impairment to Alzheimer’s disease. J. Alzheimer’s Dis..

